# Mice Lacking the Matrilin Family of Extracellular Matrix Proteins Develop Mild Skeletal Abnormalities and Are Susceptible to Age-Associated Osteoarthritis

**DOI:** 10.3390/ijms21020666

**Published:** 2020-01-19

**Authors:** Ping Li, Lutz Fleischhauer, Claudia Nicolae, Carina Prein, Zsuzsanna Farkas, Maximilian Michael Saller, Wolf Christian Prall, Raimund Wagener, Juliane Heilig, Anja Niehoff, Hauke Clausen-Schaumann, Paolo Alberton, Attila Aszodi

**Affiliations:** 1 Experimental Surgery and Regenerative Medicine (ExperiMed), Department of General, Trauma and Reconstructive Surgery, Munich University Hospital, Ludwig-Maximilians-University, 80336 Munich, Germany; Ping.Li@med.uni-muenchen.de (P.L.); lutz.fleischhauer@hm.edu (L.F.); carina.prein@uwo.ca (C.P.); Zsuzsanna.Farkas@med.uni-muenchen.de (Z.F.); Maximilian.Saller@med.uni-muenchen.de (M.M.S.); Christian.Prall@med.uni-muenchen.de (W.C.P.); Paolo.Alberton@med.uni-muenchen.de (P.A.); 2 Center for Applied Tissue Engineering and Regenerative Medicine, Munich University of Applied Sciences, 80533 Munich, Germany; hauke.clausen-schaumann@hm.edu; 3 Center for NanoScience, Ludwig-Maximilians University Munich, 80799 Munich, Germany; 4 Department of Molecular Medicine, Max Planck Institute for Biochemistry, 82152 Martinsried, Germany; cnicolae@pennstatehealth.psu.edu; 5 Center for Molecular Medicine, University of Cologne, 50923 Cologne, Germany; raimund.wagener@uni-koeln.de; 6 Center for Biochemistry, Faculty of Medicine, University of Cologne, 50931 Cologne, Germany; juliane.heilig@uni-koeln.de; 7 Cologne Center for Musculoskeletal Biomechanics, Faculty of Medicine and University Hospital of Cologne, 50931 Cologne, Germany; niehoff@dshs-koeln.de; 8 Institute of Biomechanics and Orthopaedics, German Sport University Cologne, 50933 Cologne, Germany

**Keywords:** matrilin, cartilage, bone development, articular cartilage, osteoarthritis

## Abstract

Matrilins (MATN1, MATN2, MATN3 and MATN4) are adaptor proteins of the cartilage extracellular matrix (ECM), which bridge the collagen II and proteoglycan networks. In humans, dominant-negative mutations in MATN3 lead to various forms of mild chondrodysplasias. However, single or double matrilin knockout mice generated previously in our laboratory do not show an overt skeletal phenotype, suggesting compensation among the matrilin family members. The aim of our study was to establish a mouse line, which lacks all four matrilins and analyze the consequence of matrilin deficiency on endochondral bone formation and cartilage function. *Matn1-4^−/−^* mice were viable and fertile, and showed a lumbosacral transition phenotype characterized by the sacralization of the sixth lumbar vertebra. The development of the appendicular skeleton, the structure of the growth plate, chondrocyte differentiation, proliferation, and survival were normal in mutant mice. Biochemical analysis of knee cartilage demonstrated moderate alterations in the extractability of the binding partners of matrilins in *Matn1-4^−/−^* mice. Atomic force microscopy (AFM) revealed comparable compressive stiffness but higher collagen fiber diameters in the growth plate cartilage of quadruple mutant compared to wild-type mice. Importantly, *Matn1-4^−/−^* mice developed more severe spontaneous osteoarthritis at the age of 18 months, which was accompanied by changes in the biomechanical properties of the articular cartilage. Interestingly, *Matn4^−/−^* mice also developed age-associated osteoarthritis suggesting a crucial role of MATN4 in maintaining the stability of the articular cartilage. Collectively, our data provide evidence that matrilins are important to protect articular cartilage from deterioration and are involved in the specification of the vertebral column.

## 1. Introduction

Endochondral bone development is a complex process which requires the differentiation of chondrocytes and the production of a tissue-specific extracellular matrix (ECM) by forming cartilaginous templates of the future bones. Cartilage ECM provides physical support for chondrocytes maintaining the integrity and biomechanical properties of the cartilage, such as resistance against tensile strength and compressive forces. The typical transient hyaline cartilage of the developing bones and the permanent articular cartilage are composed of the heterotypic type II/IX/XI collagen fibrils and proteoglycans, mainly aggrecan, and numerous multi-domain adaptor proteins, which interconnect the two macromolecular networks. Among the perifibrillar adaptor proteins, matrilins (MATN) form a subfamily of modular, non-collagenous ECM proteins consisting of four members, namely matrilin-1, -2, -3, and -4 [1]. All matrilin members share similar structures containing one (matrilin-3) or two (matrilin-1, -3 and -4) von Willebrand factor A (VWA) domains, various numbers of epidermal growth factor (EGF) like domains and a coiled-coil (CC) α-helical oligomerization module. In mice, matrilin-1 (MATN1) and matrilin-3 (MATN3) are predominantly expressed in the developing epiphyseal and growth plate cartilages [2,3], while matrilin-2 (MATN2) and matrilin-4 (MATN4), besides cartilage, are also present in various extra-skeletal tissues [4,5]. Matrilins interacts with numerous cartilage ECM components including aggrecan (ACAN) [6], collagen II [7], collagen IX [8], cartilage oligomeric matrix protein (COMP) [9] and decorin [10], thereby may interconnect and stabilize the macromolecular networks of collagen fibrils and the aggregating proteoglycan aggrecan [1,10,11].

During skeletal development, MATN1, MATN3, and MATN4 display a largely overlapping expression pattern. These matrilins are abundant in epiphyseal and growth plate cartilage, whereas MATN2 is strongly expressed in the perichondrium/periosteum and moderately in the proliferative zone of the growth plate [12,13]. At the forming articular surface, the outermost superficial cell layers express MATN2 and MATN4, whereas the deeper cell layers of the articular surface express MATN3 and MATN4 but not MATN1 [13]. In more mature articular cartilage, all matrilins are expressed at very low levels [13].

To date, the association of a human connective tissue disorder has been only identified in the genes coding for matrilin-3 and matrilin-1. Multiple epiphyseal dysplasia (MED) is a clinically and genetically heterogeneous skeletal dysplasia characterized by joint pain and stiffness, and early onset of osteoarthritis (OA). Autosomal dominant forms of MED are caused by mutations in the genes encoding matrilin-3 (MATN3), collagen IX chains (COL9A1, COL9A2 and COL9A3) and cartilage oligomeric matrix protein (COMP) [14]. The MED mutations identified in MATN3 are missense and predominantly confined to the β-sheet regions of the VWA domain [15,16,17]. In vitro experiments suggest that these mutations lead to the retention of the mutant MATN3 in the rough endoplasmic reticulum, where it accumulates as an unfolded intermediate and activates unfolded protein response [18,19,20]. In addition to MED, mutations in MATN3 have been described in bilateral hereditary micro-epiphyseal dysplasia (BHMED) [21] and spondylo-epi-metaphyseal dysplasia (SEMD) [22] patients. Furthermore, a low occurrence of linkage of *MATN3* to hand OA and spinal disc degeneration has been reported [23,24,25]. The association of MATN1 with osteoarthritis was described in the Dutch but not in the British population [26,27]. More recently, *MATN1* was suggested as a candidate gene for idiopathic scoliosis [28] and mandibular prognathism [29], and as a genetic modifier of SEMD with joint laxity [30].

Despite the suggested integrative functions of matrilins in the cartilage ECM, ablation of matrilin genes in mice does not lead to an overt phenotype. Single knockout mice lacking matrilin-1 (*Matn1^−/−^*) or matrilin-3 (*Matn3^−/−^*) [31,32] were generated in our laboratory and these null mice showed no signs of chondrodysplasia or any other obvious skeletal phenotype. In contrast, subtle defects were identified in other matrilin mutant strains generated by independent laboratories. Abnormal collagen II fibrils and stiffness of the cartilage ECM were reported in the matrilin-1 deficient mice [33,34], while accelerated differentiation of embryonic hypertrophic chondrocytes in the growth plate, increased bone mineral density and higher incidence of knee osteoarthritis were found in matrilin-3 knockout mice [35]. Although, we were unable to detect these skeletal phenotypes in our single knockout lines, we have reported a mild increase of collagen fibrillar thickness in *Matn1^−/−^*, *Matn3^−/−^* and matrilin-1/matrilin-3 double deficient mice (*Matn1^−/−^/Matn3^−/−^*) by electron microscopy [36]. This finding implies that matrilin-1 and matrilin-3 may have only a minor role in the proper ultrastructural organization of collagen network in cartilage. Interestingly, matrilin-2 and matrilin-4 deficient mice, which also develop without obvious skeletal abnormalities [12,37], instead present extra-skeletal phenotypes. *Matn2^−/−^* mice display impaired functional recovery after femoral nerve lesion, indicating an essential role of matrilin-2 for peripheral nerve regeneration [38], while *Matn4^−/−^* mice show increased proliferation of hematopoietic stem cells upon myelosuppressive chemotherapy, inflammatory stress and transplantation [37].

The similar structure, function, and expression pattern of matrilins suggest compensation among the family members. Indeed, we previously demonstrated that matrilin-4 is up-regulated in the cartilage of *Matn1^−/−^* and *Matn1^−/−^/Matn3^−/−^* mice, providing the first experimental evidence that biochemical compensation could exist between matrilins in vivo [36]. In order to further extend our knowledge about the skeletal function of matrilins, herein we report the analysis of mice lacking all matrilins. Quadruple mutant mice (*Matn1-4^−/−^*) have a reduced number of lumbar vertebrae due to lumbosacral homeotic transition and osteoarthritic-like degeneration develops in mice older than 18 months. Interestingly, similar articular cartilage degeneration was observed in aged matrilin-4 deficient mice, indicating an unexpected role of matrilin-4 in protecting articular cartilage from age-associated, spontaneous osteoarthritis.

## 2. Results

### 2.1. Biochemical Compensation in Cartilage of Mice Lacking Matrilins

**Figure 1:**
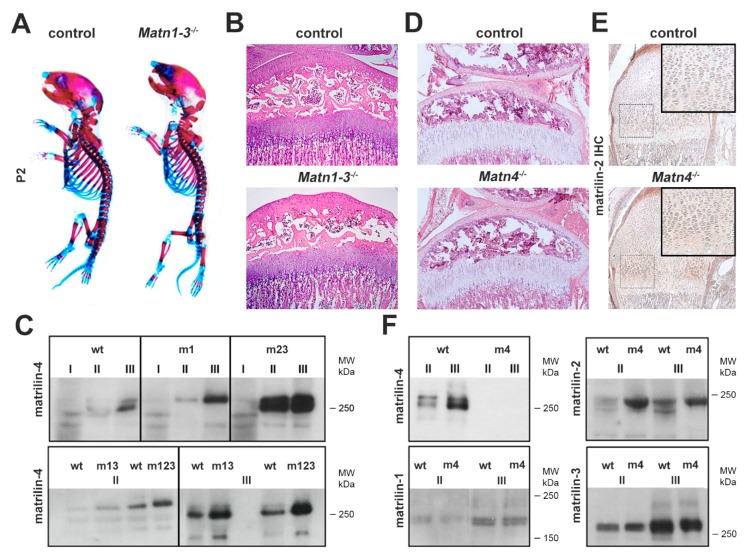
Biochemical compensation among matrilins. (**A**) Whole-mount skeletal staining at postnatal day 2 (P2) shows no obvious skeletal defects in mice lacking matrilin-1, -2 and -3 (*Matn1-3^−/−^*) compared to wild type. (**B**) HE staining of the proximal tibia (original magnification × 10) of the knee joint indicates normal growth plate and articular cartilage structures in *Matn1-3^−/−^* mice at 4 weeks of age. (**C**) Western blot analyses of sequential cartilage extracts (I-neutral salt; II-high salt/EDTA; III-GuHCl) from newborn mice indicates upregulation of matrilin-4 in mice lacking matrilin-1 (m1), matrilin-2 and -3 (m23), matrilin-1 and -3 (m13), and matrilin-1, -2, and -3 (m123). (**D**) HE staining of the proximal tibia (original magnification × 10) of the knee joint at 4 weeks of age demonstrates that mice lacking matrilin-4 (*Matn4^−/−^*) have a normal structure of long bones. (**E**) Immunohistochemistry (IHC, original magnification × 10) indicates the increased deposition of matrilin-2 in the growth plate of the humerus (rectangle, original magnification × 20) of *Matn4^−/−^* mice. (**F**) Western blot analyses show increased amounts of matrilin-2 in cartilage extracts of *Matn4^−/−^* mice (m4), while the levels of matrilin-1 and matrilin-3 are unchanged. Abbreviation: MW-molecular weight marker.

Previously we have demonstrated that knockout mice lacking matrilin-1 (*Matn1^−/−^*), matrilin-3 (*Matn3^−/−^*) or both matrilin-1 and matrilin-3 (*Matn1^−/−^/Matn3^−/−^*) exhibit only mild ultrastructural abnormalities of the collagenous fibrillar network of the cartilage without manifestation of any obvious skeletal defects [31,32,36]. We have also found, however, that matrilin-4 (MATN4) is upregulated in *Matn1^−/−^* and *Matn1^−/−^/Matn3^−/−^* mice but not in *Matn3^−/−^* mice in knee cartilage tissues sequentially extracted with high salt containing 10 mM ethylenediaminetetraacetic acid (EDTA) (fraction II) and 4 M guanidine hydrochloride (GuHCl) (fraction III), while matrilin-2 (MATN2) was deposited normally in those mutants compared with controls [36]. In the present study, we have analyzed further compound knockout mice lacking MATN1, MATN2 and MATN3 in various combinations (*Matn2^−/−^/Matn3^−/−^*; *Matn1-3^−/−^)*, and we could confirm that the homotrimeric form of MATN4 was consistently upregulated in fractions II and III, but not in neutral salt extracts (fraction I) of animals lacking MATN1 and/or MATN2 (Figure 1C). MATN4 compensatory upregulation was especially prominent in mice, which lacked MATN2 in addition to MATN1 and/or MATN3, such as *Matn2^−/−^/Matn3^−/−^* and *Matn1-3^−/−^* mice. Importantly, these multiple knockouts including the triple mutant *Matn1-3^−/−^* mice had normal gross skeleton and displayed normal growth plate and articular cartilage histoarchitectures at birth and at various postnatal stages (Figure 1A,B and not shown). Similarly, mice lacking MATN4 developed a normal skeleton without apparent abnormalities of the zonal and columnar structure of the cartilaginous growth plate of the long bones (Figure 1D). Interestingly, immunohistochemical staining revealed an upregulation of MATN2 deposition in the proliferative and hypertrophic zones of the newborn growth plate cartilage in *Matn4^−/−^* mice (Figure 1E). Using Western blots, we could confirm stronger signals for MATN2 in fractions II/III of matrilin-4 mutant cartilage extracts compared with wild type, while the levels of MATN1 and MATN3 did not change significantly (Figure 1F). The expression of *Matn2* at mRNA levels was comparable between control and *Matn4^−/−^* mice in newborn limb cartilage (data not shown). Collectively, our data demonstrate that MATN2 and MATN4 biochemically compensate for the lack of MATN4 and MATN1, respectively, in the newborn mouse knee cartilage.

### 2.2. Loss of Matrilins Results in Modulation of Lumbosacral Identity of the Vertebrae

**Figure 2:**
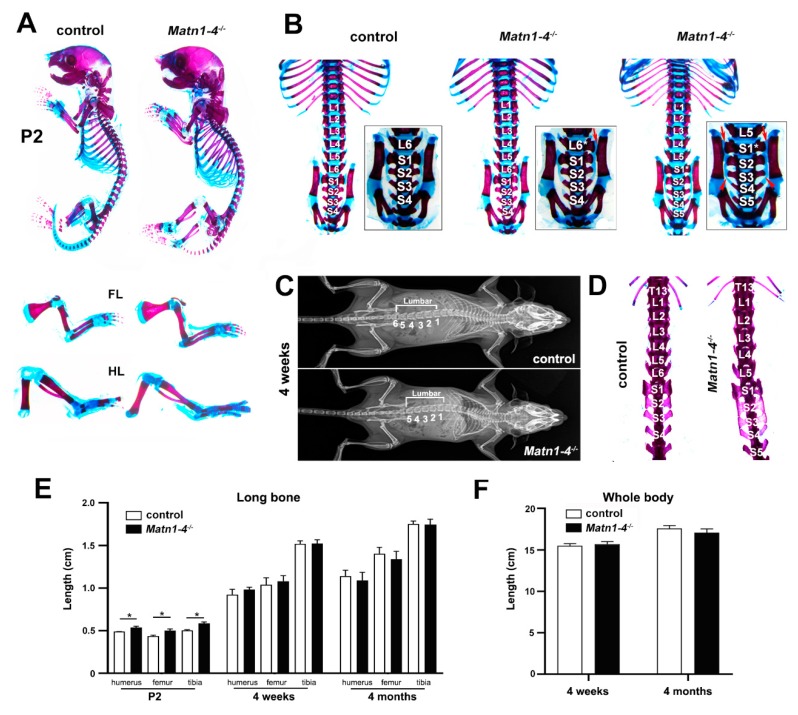
Skeletal phenotype in mice lacking all matrilins. (**A**) Skeletal staining at P2 showed normal formation of the elements of the appendicular skeleton. (**B**–**D**) Skeletal staining and X-ray analyses at P2 and 4 weeks of age revealed a highly penetrant homeotic transformation at the lumbar-sacral border of the axial skeleton. (**B**) Wild-type (control) mice have 6 lumbar (L1 to L6) and 4 sacral (S1 to S4) vertebrae, S1 articulates to the ilium and S1-S2-S3 are fused. In *Matn1-4^−/−^* mice, L6 is sacralized (S1*) resulting in 5 lumbar (L1 to L5) and 5 sacral vertebrae (S1 to S5). In the mutants, the sacralized L6 vertebra (S1*) gained the typical S1 wing shape (red arrows on B), S1* and S2 are articulate to the ilium and S1*/S2/S3/S4 are fused. The arrowheads depict the fusion between S3 and S4. The heterozygous offspring exhibit an intermediate pattern with only one side of L6 is sacralized (L6*) by gaining a wing-shaped transverse process and articulating to the ileum (L6*, red arrow), while the other side retained the lumbar identity. (**C**) Representative X-ray images of wild-type and *Matn1-4^−/−^* mice at 4 weeks. (**D**) Skeletal preparations at 4 weeks demonstrate the sacralization of L6 in the quadruple KO mice. (**E**) Measurements of the lengths of the appendicular skeletal elements indicate a moderate but significantly increased size of mutant long bones at P2. At 4 weeks and 4 months, there is no significant difference between the genotypes. Statistical significance calculated by Mann-Whitney *U* test where * *p* < 0.05. (**F**) Comparable body length of wild-type and *Matn1-4^−/−^* animals at 4 weeks and 4 months of age. Abbreviations: FL-forelimb; HL-hindlimb.

In order to assess the role of matrilins in skeletal development, we have generated quadruple knockout mice lacking all members of the protein family (*Matn1-4^−/−^*). Homozygous mutant breeding revealed normal litter size with offspring, which had normal life span and developed no apparent gross abnormalities. However, we have noticed by regular inspection of the cages, that *Matn1-4^−/−^* mice showed reduced fear and anxiety when they were picked up by the tail and, in general, were physically less active and motile in the cage compared with control mice. In this study, we have focused on the skeletal analysis of the mice, therefore, the behavioural abnormalities were not investigated further. Alcian blue and alizarin red double whole-mount skeletal staining of mutant and wild-type (control) mice at postnatal day 2 (P2) showed normal formation of the elements of the appendicular skeleton (Figure 2A). Closer inspection of the long bones on skeletal preparations or X-ray micrographs demonstrated very moderate but significantly increased lengths of the tibia, femur, and the humerus at P2 in the *Matn1-4^−/−^* mice compared with control mice (*p* < 0.05). However, the lengths of these skeletal elements were comparable at the ages of four weeks and four months (Figure 2A,C,E). Similarly, the whole body length (the distance between the nose and the tip of the tail) was comparable between wild-type and *Matn1-4^−/−^* animals at four weeks and four months of age (Figure 2F).

In contrast to the appendicular skeleton, skeletal staining and X-ray analyses revealed a highly penetrant homeotic transformation at the lumbar-sacral border of the axial skeleton (Figure 2B–D). The mouse axial skeleton typically consists of 30 pre-caudal vertebrae including 7 cervical (C), 13 thoracic (T), 6 lumbar (L) and 4 sacral (S) ones giving the formulation of C7/T13/L6/S4 [39,40]. We found that 87.5% of *Matn1-4^−/−^* mice have five lumbar and five sacral vertebrae (C7/T13/L5/S5), while only 6.7% of wild-type animals presented this vertebral pattern (Figure 2B,C and Table 1). Apparently, the missing L6 vertebrae in the *Matn1-4^−/−^* mice gained a sacral identity (S1* in Figure 2B,D) resulting a sacral pattern of S1*/S2/S3/S4/S5. In control, S1 articulates to the ilium, S1/S2 acquire wing-shaped transverse processes and S1/S2/S3 are fused. In the mutants, the sacralized L6 vertebra (S1*) and the true S1 (now S2) articulate to the ileum (red arrows on Figure 2B), S1*/S2/S3 have wing-shaped transverse processes and S1*/S2/S3/S4 are fused. Interestingly, when a *Matn1-4^−/−^* male was crossed with a C57/BL6 female, the heterozygous offspring exhibited either the normal L6/S4 pattern (three out of seven mice), the homeotic transformed L5/S5 pattern (two out of seven) or an intermediate, asymmetric L6*/S4 pattern (two out of seven mice). In the latter case, only one side of L6 is sacralized (L6*) by gaining a wing-shaped transverse process and articulating to the ileum (Figure 2B, *Matn1-4^+/−^*, L6*, arrows), while the other side retained the lumbar identity.

### 2.3. Lack of Matrilins Has No Adverse Effect on Structural and Functional Properties of the Cartilaginous Growth Plate in Long Bones

**Figure 3:**
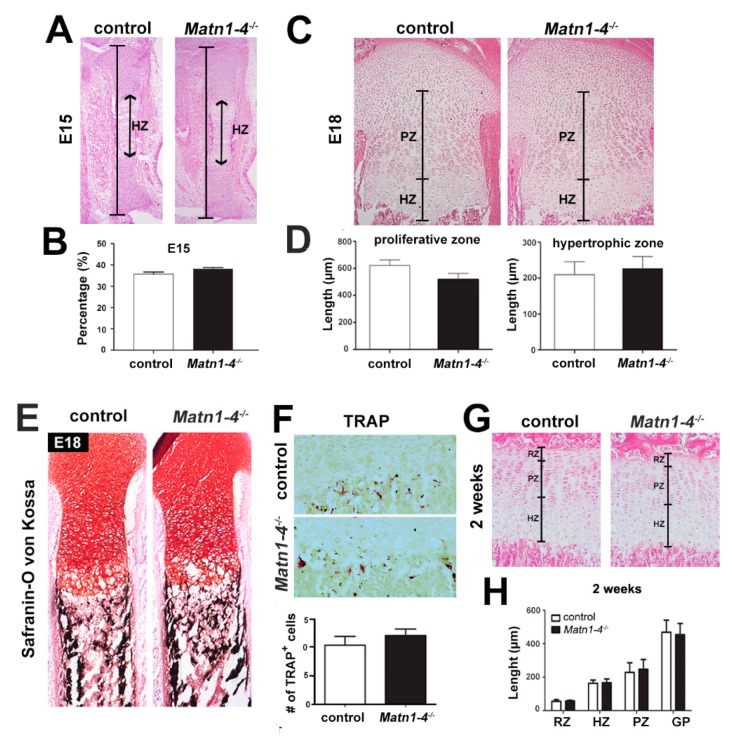
Normal cartilage development and growth plate structure in long bones of *Matn1-4^−/−^* mice. (**A**) HE-stained proximal tibiae at E15.5 show normal length, structure and hypertrophic zone (HZ) in *Matn1-4^−/−^* mice. (**B**) Percentage of the hypertrophic zone relative to the entire cartilaginous mass of the tibia was similar in *Matn1-4^−/−^* and in the control animals. (**C**) HE-stained proximal tibiae at E18 and morphometric measurements of the proliferative (PZ) and hypertrophic (HZ) zones (**D**) demonstrate normal structure of the growth plate in the *Matn1-4^−/−^* mice. Safranin O-von Kossa (**E**) and TRAP staining (**F**) show comparable mineralization and chondroclast/osteoclast activity at the chondro-osseous junction in control and *Matn1-4^−/−^* mice. Quantification of the TRAP positive cells in the tibial growth plate of control and *Matn1-4^−/−^* mice shows no difference at the chondro-osseous junction. (**G**) HE-staining of the tibial growth plate at 2 weeks and morphometric analysis (**H**) of the length of the entire growth plate (GP) and the separated growth plate zones (RZ-resting; PL-proliferative; HZ-hypertrophic) indicate normal columnar organization of the chondrocytes and normal GP zonality in *Matn1-4^−/−^* mice. Original magnifications: ×10 for (**A**), (**C**) and (**E**); ×20 for (**F**) and (**G**).

Next, we examined long bone development by histological tools at various developmental stages. Hematoxylin and eosin (HE) staining of the hindlimb at embryonic day 15 (E15) demonstrated that the length of the whole tibia and percentage of the hypertrophic zone relative to the entire cartilaginous mass were similar in *Matn1-4^−/−^* and control animals (Figure 3A,B). At E18, the proximal tibia exhibited comparable columnar organization of the growth plate (Figure 3C) and similar length of the proliferative and hypertrophic zones in control and *Matn1-4^−^*^/*−*^ mice (Figure 3D). Similarly, the mineralization of the primary ossification center, judged by Safranin orange-von Kossa staining (Figure 3E), and the resorption at the chondro-osseous junction, visualized by tartrate resistant acid phosphatase (TRAP) activity staining (Figure 3F), were indistinguishable between control and quadruple mutant animals. At postnatal stage two weeks, the columnar organization of the growth plate was normal, and morphometric measurements of the lengths of the resting, proliferative and hypertrophic zones and the total growth plate showed no statistically significant difference between control and *Matn1-4^−/−^* mice (Figure 3G,H).

**Figure 4:**
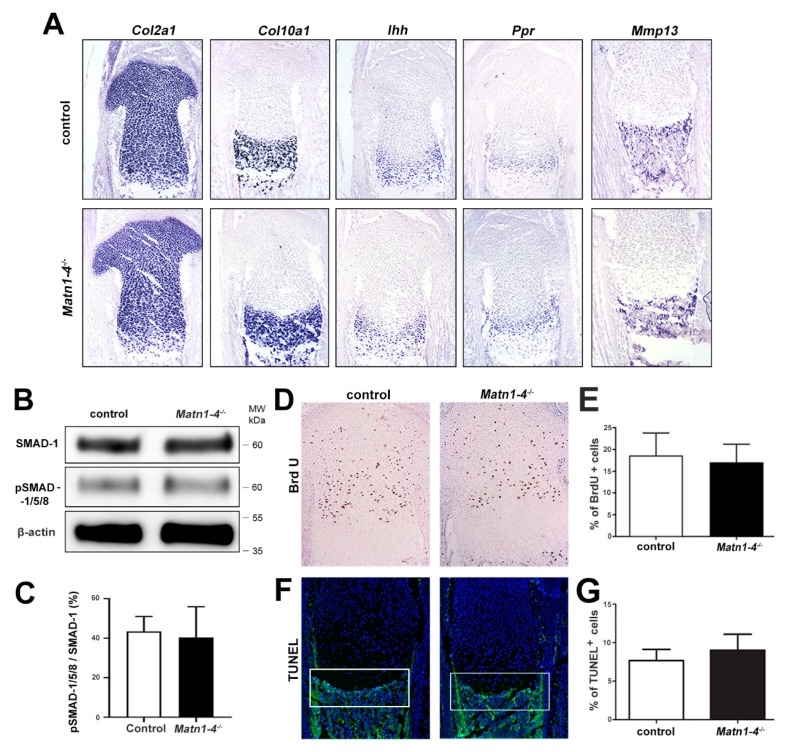
Chondrocyte differentiation, proliferation and apoptosis are not altered in *Matn1-4^−/−^* mice. (**A**) Non-radioactive in situ hybridization for collagen II (*Col2a1*), collagen X (*Col10a1*), indian hedgehog (*Ihh*), PTH/PTHrP receptor (*Ppr*) and matrix metalloproteinase-13 (*Mmp13*) show similar expression pattern in newborn control and mutant mice. Western blotting (**B**) and densitometric quantification (**C**) indicate normal activation of SMAD-1/5/8 in mutant primary chondrocytes. (**D**) BrdU incorporation assay and quantification (**E**) at the newborn stage indicate normal chondrocyte proliferation in the *Matn1-4^−/−^* growth plate. TUNEL staining (**F**) and quantification (**G**) do not reveal any difference in cell death at the chondro-osseous junctions (white boxes) in newborn control and *Matn1-4^−/−^* mice. Abbreviation: MW-molecular weight marker. Original magnifications: ×10 for (**A**), (**D**) and (**F**).

Chondrocyte differentiation was investigated in the growth plate of newborn tibia by in situ hybridization. We found no difference in the expression domains of the typical differentiation markers between control and *Matn1-4^−/−^* mice (Figure 4A). It has been previously suggested that matrilin-3 inhibits chondrocyte hypertrophy by suppressing BMP-2/SMAD-1 signaling [41]. Therefore, we have investigated the activation of SMADs in protein extracts of primary mouse chondrocytes isolated from newborn rib cages using a phospho-specific antibody. Western blotting displayed similar phospho-SMAD-1/5/8 levels between the genotypes (Figure 4B,C), arguing against significantly altered BMP-2 signaling in cartilage lacking matrilins.

Next, we analyzed the proliferation and survival of the chondrocytes in the tibial growth plate. Proliferation was assessed by bromodeoxyuridine (BrdU) incorporation assays, which revealed a similar proliferation rate in control and *Matn1-4^−/−^* mice at the newborn stage (Figure 4D,E) or at four and eight weeks of age (data not shown). We further investigated cell death by terminal deoxynucleotidyl transferase dUTP nick end labeling (TUNEL) assay in newborn samples and found comparable numbers of apoptotic chondrocytes at the chondro-osseous junctions in both control and quadruple mutant mice (Figure 4F,G).

Collectively, the data above demonstrate that chondrocyte differentiation, proliferation, and survival occur normally in the growth plate of long bones in mice lacking all matrilins.

### 2.4. Normal Deposition but Altered Extractability of Binding Partners in the Cartilage ECM of Quadruple Knockout Mice

**Figure 5:**
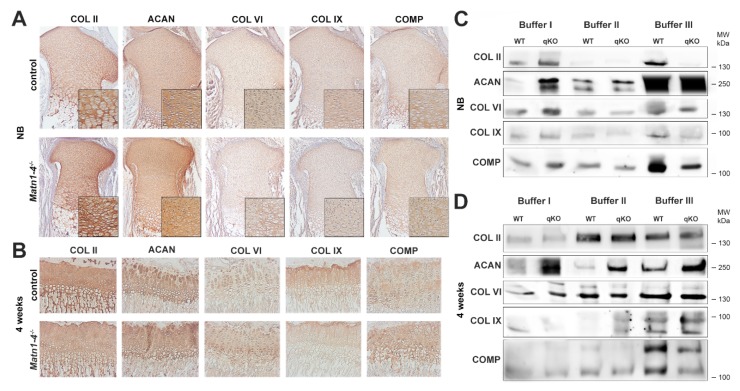
Immunohistochemical and biochemical analysis of the deposition and solubility of the binding partners of matrilins. Immunohistochemistry at newborn (**A**) and 4 weeks (**B**) stages indicates no apparent differences in the deposition of collagen II (COL II), collagen VI (COL VI), collagen IX (COL IX), aggrecan (ACAN) and cartilage oligomeric matrix protein (COMP) between control and *Matn1-4^−/−^* mice. Original magnifications: × 10 for (**A**) and (**B**); x 20 for inserts in (**A**). Western blot analysis of cartilage extracts in newborn (**C**) and 4 weeks old (**D**) animals. In newborn, slightly increased extractability of collagen II, collagen IX, aggrecan, collagen VI and COMP in buffer I and significantly weaker signals for collagen II, collagen IX and COMP were detected in fraction III isolated from the cartilage of quadruple knockout mice compared with controls (**C**). At 4 weeks, the amount of aggrecan is increased in all fractions, the amount of collagen IX is slightly increased in fraction II and the amount of COMP is moderately decreased in fraction III in M *Matn1-4^−/−^* mice compared with the control (**D**). Abbreviation: MW-molecular weight marker.

As matrilins are adaptor proteins of the cartilage ECM interacting with aggrecan, COMP, collagen fibrils, and small leucine-rich proteoglycans (e.g., biglycan and decorin) [1], we have investigated the expression and anchorage of some of these binding partners by immunohistochemistry and biochemical analysis. Immunostaining of the proximal tibia in newborn or four-week old limbs indicated no apparent differences in the deposition of collagen II, collagen VI, collagen IX, aggrecan and COMP between control and *Matn1-4^−/−^* mice (Figure 5A,B). Western blot analysis of sequential extracts of knee cartilage isolated from newborn animals showed slightly increased extractability of collagen II, collagen IX, aggrecan, collagen VI and COMP in fraction I (neutral salt) of *Matn1-4^−/−^* mice, when compared to wild-type controls. While the amount of matrilin interacting proteins did not change in fraction II (high salt with 10 mM EDTA), weaker signals for collagen II, collagen IX, and COMP were detected in fraction III (4 M GuHCl) isolated from the cartilage of quadruple knockout mice compared with controls (Figure 5C). At four weeks, the amount of aggrecan increased in all fractions, the amount of collagen IX slightly increased in fraction II, and the amount of COMP moderately decreased in fraction III in *Matn1-4^−/−^* mice compared to control (Figure 5D).

**Figure 6:**
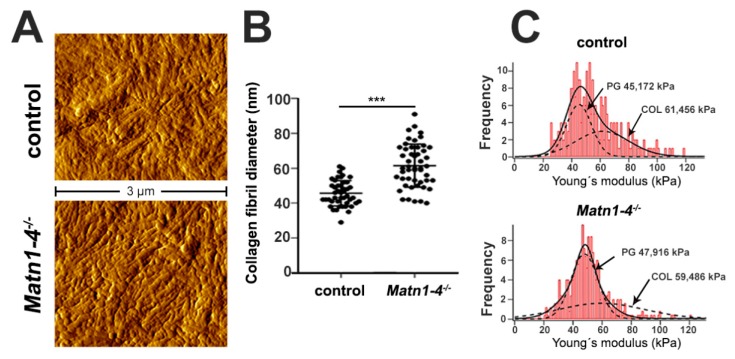
Normal growth plate cartilage stiffness but altered fibril diameter in *Matn1-4^−/−^* mice. (**A**) At 2 weeks of age, high-resolution AFM images on tibial sections show comparable organization of the collagenous networks in the interterritorial matrix in control and the mutant animals. (**B**) Quantification of the fibrillar diameter demonstrates significant thickening of the collagen fibrils in the *Matn1-4^−/−^* mice (*** *p* < 0.001). (**C**) Histograms depicting a comparable, bimodal stiffness distribution between the genotypes determined by nano-scale AFM indentation. On each histogram, the solid line represents the sum of two Gaussian functions, whereas the dashed lines indicate individual fits representing proteoglycans (first peak, PG) and the collagen fibrils (second peak, COL).

To assess the consequence of the lack of matrilins on the ultrastructural and biomechanical properties of the cartilaginous ECM, we applied atomic force microscopy (AFM) (Figure 6). We investigated the proliferative zone of the growth plate at two weeks of age on non-fixed, native sections and recorded high resolution images of the interterritorial matrix (ITM) representing the intercolumnar areas. Topographical images revealed an elaborated network of collagen fibrils in both control and *Matn1-4^−/−^* mice (Figure 6A). Quantification of the diameter of heterotypic collagen fibrils revealed a significantly increased thickness of the fibers (Figure 6B), similar to the results reported earlier in *Matn1^−/−^/Matn3^−/−^* mice [36]. The mean fibril diameter was 45.64 ± 7.29 nm in control and 61.50 ± 12.38 nm in *Matn1-4^−/−^* mice (*p* < 0.001). Interestingly, nanoindentation measurements indicated comparable compressive stiffness of the ITM. The frequency of the elastic moduli showed a bimodal distribution with a first peak (representing the proteoglycan moiety) at 45.17 ± 0.93 kPa in controls and at 47.91 ± 0.34 kPa in quadruple mutants, and with a second peak (representing the collagen fibrils) at 61.46 ± 7.79 kPa in controls and 59.48 ± 2.14 kPa in *Matn1-4^−/−^* mice (Figure 6C).

Taken together, although the expression and distribution of the analyzed matrilin binding partners were not obviously altered in the growth plate cartilage of *Matn1-4^−/−^* mice, the solubility of these proteins in the ECM is moderately affected by the loss of all matrilins. Despite this mild biochemical phenotype and the increased diameter of collagen fibrils, the biomechanical behavior of the cartilage ECM, characterized by the compressive stiffness, is apparently not affected in the growth plate of adolescent *Matn1-4^−/−^* mice.

### 2.5. Depletion of All Matrilins in the Articular Cartilage Leads to Severe Spontaneous Osteoarthritis in Mice

**Figure 7:**
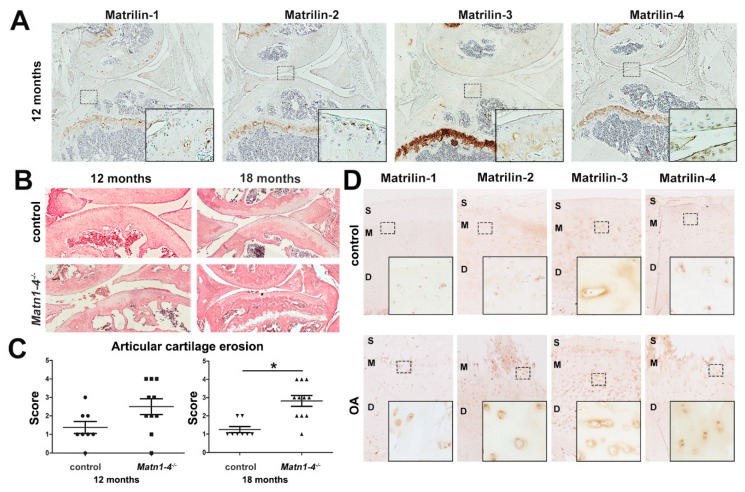
*Matn1-4^−/−^* mice develop age-associated osteoarthritis. (**A**) Immunohistochemistry of knee joints demonstrates the moderate expression of matrilins in the articular cartilage of wild-type mice at 12 months of age. All matrilins have strong expression in the growth plate cartilage. Original magnifications: × 5 for overview pictures and x 20 for inserts. (**B**) HE staining of the knee joint at 12 and 18 months old control and *Matn1-4^−/−^* mice. Original magnification × 10. (**C**) Histological grading for cartilage degradation indicates higher incidence and severity of osteoarthritic-like erosion in *Matn1-4^−/−^* mice compared to age-matched controls (* *p* < 0.05). (**D**) Analyzing the expression of matrilins in healthy (control) and osteoarthritic human knee articular cartilage derived from the tibia by immunohistochemistry demonstrates upregulation of all matrilins in the OA tissue. The depicted zones: S-superficial; M-middle and D-deep. Original magnifications: ×5 for overview pictures and ×20 for inserts.

Next, we analyzed the consequence of the ablation of matrilin genes on age-associated changes of the articular cartilage. All matrilins are expressed in low amounts in peripheral articular cartilage areas of the knee joint in wild-type mice at one year of age (Figure 7A) [13]. We examined articular cartilage degeneration on HE-stained sections of control and *Matn1-4^−/−^* knee joints at six, 12, and 18 months of age. We found no difference in articular cartilage degradation between the genotypes at six months of age (data not shown), however, histological signs of articular cartilage damage were observed in *Matn1-4^−/−^* mice at 12 and 18 months of age (Figure 7B). Applying a scoring system for articular cartilage degradation, ranging from normal appearance (score 0) to exposure of the subchondral bone (score 5) [36], the mean histological score was 1.4 for control (*n* = 8) and 2.5 for *Matn1-4^−/−^* mice (*n* = 10) at 12 months of age. At 18 months of age, the mean histology score was 1.3 for control (*n* = 8) and 2.7 for mutant mice (*n* = 11) (*p* < 0.05) (Figure 7C). These results demonstrate that the lack of the complete matrilin family in cartilage leads to an osteoarthritic-like phenotype in aging mice from one year on, and strongly suggest that matrilins are protective for spontaneous osteoarthritis.

Analyzing the expression of matrilins in healthy and osteoarthritic human knee cartilage by immunohistochemical staining further supported the participation of matrilins in OA progression (Figure 7D). In normal human articular cartilage, matrilins are weakly expressed in the middle and deep zones: MATN1 is localized to the nucleus; MATN2 and MATN4 are associated with the cytoplasm and diffusely with the ECM; while MATN3 displays mainly pericellular/territorial matrix deposition. All matrilins are upregulated in human OA cartilage samples with severe articular cartilage degeneration (Figure 7D), which may implicate an attempt of repair by enhancing the expression of matrilin family members in the diseased ECM.

**Figure 8:**
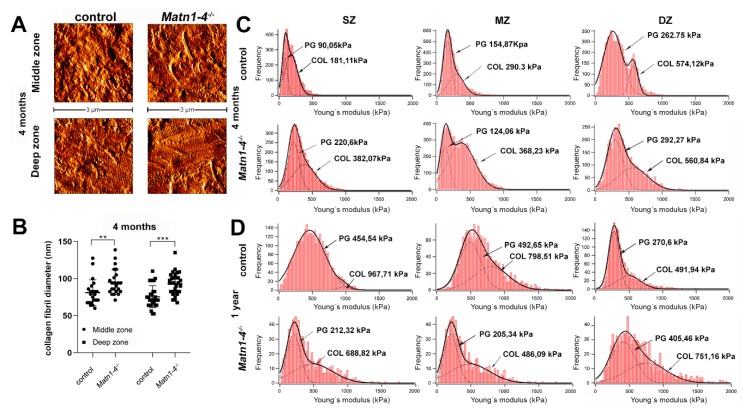
Altered biomechanical properties of articular cartilage in *Matn1-4^−/−^* mice. High resolution AFM images of articular cartilage at 4 months (**A**) showed comparable organization of the collagenous networks in control and mutant. (**B**) Significant thickening of the collagen fibrils was detected in the middle (** *p* < 0.01) and the deep zones (*** *p* < 0.001) of the articular cartilage of *Matn1-4^−/−^* mice. (**C**) Nanoindentation AFM demonstrated stiffer mutant matrix in the superficial zone (SZ) at four months. (**D**) At 1 year, softer ECM was detected in the superficial and middle (MZ) zones, whereas a stiffer matrix was observed in the deep zone (DZ) of the *Matn1-4^−/−^* mice. On each histogram, the solid line represents the sum of two Gaussian functions, whereas the dashed lines indicate individual fits representing proteoglycans (first peak, PG) and the collagen fibrils (second peak, COL).

The increased severity of spontaneous osteoarthritis in the *Matn1-4^−/−^* mice might be a consequence of the compromised biomechanical properties of the articular cartilage ECM. Therefore, we have investigated the topography and stiffness of the different articular cartilage zones by AFM on native tissue sections in quadruple mutant and control animals. High-resolution images at four months depicted the well-formed, striated collagen fibrillar network in both genotypes (Figure 8A). Quantification of the collagen fibril diameters revealed fibril thickening in the *Matn1-4^−/−^* mice (Figure 8B) supporting the findings observed in the growth plate collagen fibrils. In the middle zone, the mean fibrillar diameter was 81.07 ± 17.23 nm in control and 95.31 ± 16.76 nm in *Matn1-4^−/−^* mice (*p* < 0.01). In the deep zone, the mean fibrillar diameter was 75.52 ± 14.88 nm in control and 93.22 ± 14.71 nm in *Matn1-4^−/−^* mice (*p* < 0.001). AFM nanoindentation in the superficial, middle and deep zones of the articular cartilage showed bimodal stiffness distribution for proteoglycans and collagen fibrils. At four months of age, we observed a stiffer superficial zone in the mutant compared with wild type, while the elastic moduli in the middle and deep zone were not changed significantly (Figure 8C). At 12 months, in contrast, both peaks of the elastic moduli indicated softer superficial and middle zones in the quadruple knockout mice compared to control (Figure 8D). Interestingly however, the deep zone was stiffer in the mutant articular cartilage than in the control (Figure 8D).

**Figure 9:**
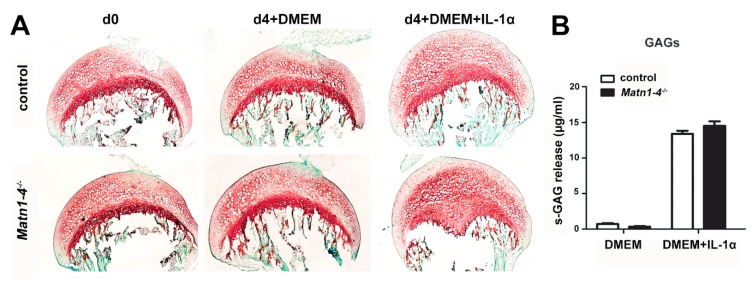
Matrilins are not involved in the interleukin-1-mediated proteoglycan loss of the articular cartilage. (**A**) Safranin-O and Fast Green staining of hip explants after culturing for 4 days (d4) with or without 10 ng/mL IL-1α. Note the similar loss of proteoglycans at the articular cartilage surface after IL-1α stimulation in control and *Matn1-4^−/−^* mice. (**B**) Quantification of the sulfated GAG release into the medium shows no difference after IL-1α stimulation between the genotypes.

To investigate whether matrilins may play a role in inflammation-mediated articular cartilage degradation, we performed an ex vivo explant culture experiment in which femoral heads were subjected or not to the influence of the pro-inflammatory cytokine interleukin-1 alpha (IL-1α). After four days in culture, the explants were investigated for proteoglycan loss by Safranin O staining on histological sections and for sulfated glycosaminoglycan (GAG) release into the medium (Figure 9A,B). Both the histochemical staining and the GAG-release assay indicated no difference in IL-1α—induced proteoglycan loss between the genotypes.

### 2.6. MATN4-Deficiency Also Enhances Age-Associated Articular Cartilage Degradation

**Figure 10:**
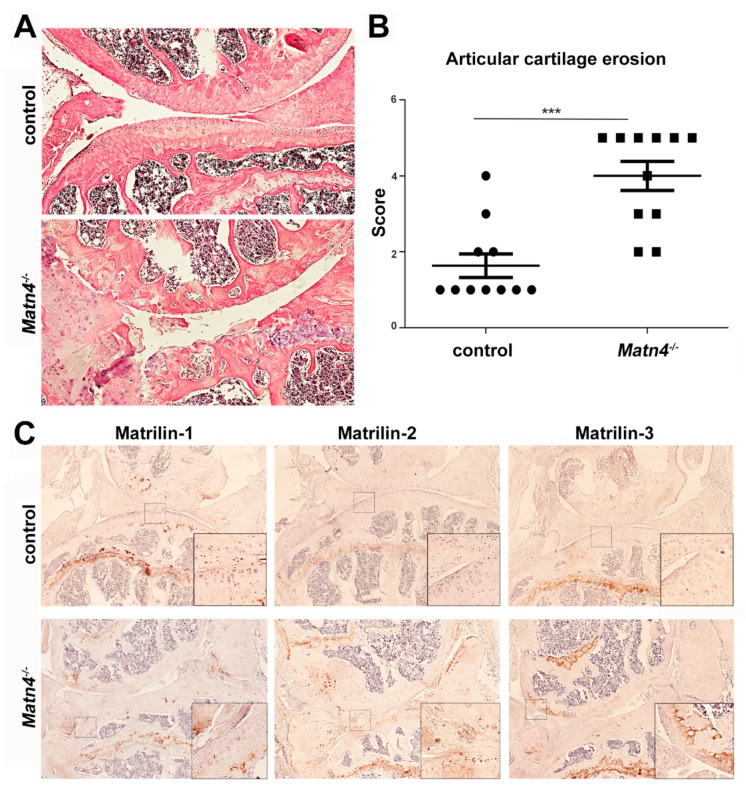
The lack of matrilin-4 leads to age-associated, spontaneous osteoarthritis of the knee joint. (**A**) HE-stained *Matn4^−/−^* knee joint (original magnification × 10) at 24 months of age exhibited severe osteoarthritis-like phenotype and (**B**) a significantly higher articular cartilage degeneration score compared to wild type (*** *p* < 0.001). (**C**) Immunohistochemistry demonstrates upregulated expression of MATN2 and MATN3 in MATN4-deficient articular cartilage. Original magnifications: ×10 for overview pictures and ×20 for inserts.

The development of spontaneous osteoarthritis in aged *Matn1-4^−/−^* mice raised the question about the contribution of the individual matrilins to the phenotype. Previously, we had not observed age-associated osteoarthritis in single MATN1, MATN2, MATN3, or double MATN1/MATN3 deficient mice [12,31,32,36]. However, the MATN4 knockout mice (*Matn4^−/−^)* have not been yet analyzed for articular cartilage degeneration. Thus, we investigated the pathology of the knee joint in 18–24 month old Matn4^−/−^ mice on HE-stained histological sections (Figure 10). *Matn4^−/−^* animals, similar to the *Matn1-4^−/−^* mice, exhibited severe osteoarthritis-like phenotype at the age of 24 months often leading to complete erosion of the articular cartilage and exposure of the subchondral bone (Figure 10A). We found that the mean articular cartilage degeneration score was 1.70 in wild-type mice and was 3.67 in the *Matn4^−/−^* mice (*p* < 0.001) (Figure 10B), implying that MATN4 may protect against osteoarthritis. Interestingly, immunohistochemistry demonstrated that the deposition of MATN2 and MATN3, but not the expression of MATN1, is upregulated in the articular cartilage of the knee joint of aged mice (Figure 10C).

## 3. Discussion

Cartilage extracellular matrix consists of macromolecular suprastructures containing the aggregates of aggrecan and hyaluronan, which are embedded into the network of collagen types II/IX/XI fibrils [42,43]. In order to fulfil biological and biomechanical functions, these macromolecular assemblies are interconnected and stabilized by various adaptor proteins including the FACIT (fibril associated collagen with interrupted triple helices) collagen type IX, family members of thrombospondins (e.g., COMP or thrombospondin-5), small leucine-rich repeat proteoglycans (e.g., decorin and biglycan) and matrilins [8,10,11]. Matrilins are von Willebrand factor A domain containing multi-subunit adaptors with prominent expression in the skeleton, especially in the cartilage [1,13]. All matrilins (MATN1, MATN2, MATN3 and MATN4) are expressed in the growth plate and the articular surface suggesting important roles for both developing, transient and permanent cartilages [1,13]. Despite the anticipated importance of matrilins in the cartilaginous skeleton, no or relatively mild cartilage abnormalities have been reported in knockout mice carrying single or double deletions for matrilin genes, suggesting functional redundancy among the family members [12,32,33,36]. In this study, we have generated and analyzed further single and compound matrilin-deficient mouse lines. We found evidence for biochemical compensation within the matrilin family, which showed that matrilins are important for patterning of the vertebral column and they, especially MATN-4, protect articular cartilage against spontaneous, age-associated osteoarthritis.

Sequential extraction of cartilage tissue is a well-established biochemical tool to assess the protein expression and ECM anchorage of matrilins, and to test their possible compensatory regulations [44]. MATN1 and MATN3 are largely insoluble and require strongly denaturing agent, like 4M GuHCl (buffer III), to be extracted from the tissue, while MATN2 and MATN4 can be partially extracted by milder buffers (e.g., high salt/EDTA, buffer II) [36,44]. We have previously shown by immunoblotting [36] and reproduced in this study (Figure 1C) that MATN4 is upregulated in buffers II and III in mouse strains lacking the matrilin-1 protein (*Matn1^−/−^* and *Matn1^−/−^/Matn3^−/−^*). We found even higher level of MATN4 upregulation when MATN2 was additionally missing in the cartilage of *Matn2^−/−^/Matn3^−/−^* and *Matn1-3^−/−^* mice (Figure 1C). Numerous non-skeletal tissues co-express MATN2 and MATN4, including the brain, the eye, and the lung. Interestingly, in a previous study, we could not observe a similar upregulation of MATN4 protein in extracts of those tissues in *Matn2^−/−^* mice [12]. However, an increased level of *Matn4* mRNA was reported in damaged sciatic nerves of *Matn2^−/−^* mice compared with control [38]. As we did not find enhanced *Matn4* mRNA expression in cartilage of *Matn1^−/−^*, *Matn3^−/−^*, *Matn1^−/−^/Matn3^−/−^* [36] and *Matn1-3^−/−^* mice (data not shown), our data implicate that MATN4 may exhibit compensatory upregulation for MATN2 only at protein levels, and specifically in cartilage. Importantly, we showed in this study that only MATN2 is upregulated in cartilage of *Matn4^−/−^* mice, while the protein levels of MATN1 and MATN3 were unchanged in matrilin-4-deficient mice compared with control (Figure 1E,F). Taken together, accumulated evidence from our previous [36] and current studies demonstrate that MATN2 and MATN4 compensate each other mutually at the protein level in mouse cartilage tissue.

The prominent expression of matrilins in the developing long bones and the chondrodysplasia phenotypes associated with dominant-negative mutations of the human *MATN3* gene suggest a role of matrilins for skeletal growth. We showed previously that mice lacking MATN1, MATN2, MATN3 and MATN1/MATN3 have normal skeleton, probably due to functional redundancy of the family members [12,31,32,36]. The lack of obvious cartilage abnormalities in *Matn4^−/−^* (Figure 1D) and *Matn1-3^−/−^* mice (Figure 1A,B) further support this hypothesis, which prompted us to analyze skeletal development and cartilage functions in details in mice lacking all matrilins. Surprisingly, *Matn1-4^−/−^* mice show normal growth indicated by the comparable body weights and the similar length of the skeletal elements of the appendicular skeleton in adolescent (four weeks) and adult (four months) *Matn1-4^−/−^* mice and control mice (Figure 2A,C,E,F). However, we observed slightly but significantly increased lengths of the femur, tibia, and humerus in *Matn1-4^−/−^* mice just shortly after birth (P2), which could possibly be explained by the differences in the genetic background. All matrilin single and multiple mutants were on C57BL/6 × 129/Sv background and *Matn1-4^−/−^* mice were kept in homozygous breeding. As control, we used wild-type mice on the same, mixed genetic background. However, we could not ensure the same ratio of the C57BL/6 and the 129/Sv genetic material in control and *Matn1-4^−/−^* mice. Inbred 129/Sv mice have longer gestation length, smaller litter size, and increased body weight at birth compared to inbred C57BL/6 mice [45], which suggest that even a mild shift towards higher 129/Sv contribution in *Matn1-4^−/−^* mice compared to wild type could result in an enlarged skeleton perinatally. In accordance with our observation that the lengths of skeletal elements were normal from four weeks of age in *Matn1-4^−/−^* mice, the two inbred strains display a similar body weight at 16 weeks [46].

Importantly, careful histological assessment of the development of the tibia at various embryonic and postnatal stages did not reveal any abnormalities of chondrocyte differentiation, growth plate structure and function in *Matn1-4^−/−^* mice (Figure 3 and Figure 4). A previous in vitro study by Yang et al. showed that MATN3 binds to and inhibits BMP-2, which in turn suppresses SMAD-1/5 promoter activity, reduces SMAD-1 phosphorylation and inhibits the expression of the hypertrophic chondrocyte marker gene collagen X (*Col10a1*) in cultured chondrocytes [41]. It has been also reported that *Matn3^−/−^* mice, established in the Chen laboratory, display premature hypertrophic differentiation in the embryonic tibial growth plate at E16.5 and E17.5 [35], but not at the newborn stage, which was associated with increased SMAD1 activation in the proliferative zone of the growth plate at E18.5 [41]. Interestingly, in another study, *MATN3* induced the chondrogenesis of murine ATDC5 chondroprogenitor cells by elevating the gene expression of aggrecan (*Acan*) and collagen II (*Col2a1*), but did not alter the expression of *Col10a1* [47]. In contrast, MED or SEMD mutant *MATN3* constructs abolished *Acan* and/or *Col2a1* mRNAs expression and upregulated *Col10a1* expression by interfering with TGF-β signaling [47]. Although these studies suggest a role of MATN3 for the modulation of hypertrophic differentiation of chondrocytes, *Matn1-4^−/−^* mice, however, do not show aberrant chondrogenic differentiation. Histomorphometry at different postnatal stages (Figure 3C,D,G,H) and in situ hybridization at P2 (Figure 4A) indicated normal lengths of growth plate zones and the normal expression of chondrocyte differentiation markers, respectively. At E15.5, the ratio of the total tibial length and the length of the hypertrophic core was comparable between wild-type and *Matn1-4^−/−^* animals indicating that the hypertrophic differentiation rate is not affected by the lack of matrilins during embryonic development of long bones (Figure 3A). Furthermore, the normal proliferation rate of growth plate chondrocytes, the proper and timely formation and mineralization of the chondro-osseous junctions, and the normal columnar structure in *Matn1-4^−/−^* mice clearly indicate that matrilins are dispensable for growth plate morphogenesis.

Matrilins interact through their VWA domains with other matrix constituents, including collagens (collagen types II, IX), proteoglycans (aggrecan, biglycan, decorin) [6], and COMP [9], and they can form both collagen-dependent and collagen-independent networks [3,48,49]. A growing body of evidence suggests that the disturbance of these interacting molecular assemblies could affect the integration of ECM molecules, collagen fibril formation and cartilage mechanical conditions. Ablation of collagen IX in mice results in abnormal perinatal organization of the growth plate architecture [50,51] associated with reduced integrations of COMP, MATN1, MATN3, and MATN4 into the ECM [8,52,53,54], softer cartilage matrix [54], and increased collagen fibril diameter [8,51]. COMP deficiency mildly impacts growth plate structure [55] and moderately influences the deposition and fibrillar integration of MATN3, but has no apparent effect on collagen fibrillogenesis [8,51]. While collagen fibrils with larger diameters were also reported in *Matn1^−/−^* and *Matn1^−/−^/Matn3^−/−^* knockout mice [33,36], the solubility of the matrilin interacting partners biglycan, decorin, COMP and collagen II were not altered in the cartilage of *Matn1^−/−^/Matn3^−/−^* double deficient mice [36]. In the epiphyseal cartilage of *Matn1-4^−/−^* knockout mice, we did not observe an obvious difference in the deposition of ECM molecules such as aggrecan, COMP and collagen types II, IX, and VI using immunohistochemistry (Figure 5A,B). However, alterations in their extractability were noticed by Western blotting. At the newborn stage, when the cartilage ECM is less mature, all investigated proteins showed increased solubility in neutral salt (buffer I), while collagens II, IX and COMP were present in a clearly reduced extractable amount in chaotropic buffer containing GuHCl (buffer III) (Figure 5C). At four weeks, when the cartilage matrix is undergoing a maturation process, the amount of extractable aggrecan was increased in all fractions of the cartilage of *Matn1-4^−/−^* mice compared with that in the control, whereas the solubility of the other ECM proteins did not change significantly (Figure 5D). These results suggest that matrilins may have a matrix-stabilizing role by supporting the firm anchorage of their interaction partners into the ECM when the cartilage undergoes extensive perinatal growth. Using high-resolution AFM imaging, we found collagen fibrils with increased diameters in the growth plate cartilage of two-week old *Matn1-4^−/−^* mice (Figure 6B), confirming the established role of MATN1 and/or MATN3 in the control of lateral growth of collagen fibrils [33,36]. Nanoscale AFM indentation, however, demonstrated normal stiffness of the growth plate cartilage in *Matn1-4^−/−^* mice, which implies that matrilins are dispensable for cartilage biomechanics, at least at this stage of postnatal development.

In humans, chondrodysplasia-causing mutations in COMP, collagen IX, or MATN3 are frequently associated with premature osteoarthritis. Matrilin-3 is present at low levels in the joint articular surface and its deposition is upregulated in cartilage and in the synovial fluid of patients with OA as a consequence of cartilage degradation [56,57,58,59,60]. Studies with recombinant MATN3 and human primary chondrocytes have revealed that MATN3 exhibits a context-dependent anabolic or catabolic function by influencing the expression of pro-inflammatory cytokines, ECM degradation enzymes, and ECM synthesis [59,61,62,63] through the modulation of protein kinase B (AKT) [59], interleukin-6 [62], and interleukin-1 [63,64] signaling pathways. The neoexpression of MATN1 was also observed in the cartilage of OA or rheumatoid arthritis patients [65,66], and MATN2 was recently observed in total knee arthroplasty and suggested as a biomarker for OA [67]. In contrast to other matrilins, down-regulation of *MATN4* gene expression was reported to be associated with knee OA progression [68]. In the present study, we have performed, for the first time, a comprehensive immunohistochemical study to assess the deposition of matrilins in normal and osteoarthritic human knee articular cartilage (Figure 7D). We observed that matrilins are present at various amounts in the healthy articular cartilage, preferentially in the middle and deep zones. MATN1 shows a very weak, cell-associated staining pattern; MATN3 displays predominantly pericellular expression; MATN2 and MATN4 exhibit moderate cellular staining. In severely damaged OA cartilage, all matrilins are upregulated, indicating that each member of the matrilin family participates in the cellular response for the advanced disease as an attempt to protect the tissue from further degradation.

Indeed, the most striking phenotype of the *Matn1-4^−/−^* mice was the development of severe osteoarthritis in aged mice (Figure 7B,C). In mice, matrilins are differentially expressed in the developing and mature articular cartilage. MATN2 and MATN4 are present, whereas MATN1 and MATN3 are absent at the superficial zone of the growing epiphysis [12,13,31,69,70]. In adult joints, immunohistochemistry demonstrated a very moderate deposition of matrilins in the articular cartilage [13] (Figure 7A). Interestingly, a lineage tracing experiment, in which the matrilin-1-Cre (*Matn1-Cre*) knock-in mice were crossed with the floxed ROSA26-LacZ reporter mouse line (R26R), demonstrated the lack of beta-galactosidase signal at the joint surface, suggesting that matrilin-1 is not expressed in the mature articular cartilage [70]. The OA phenotype in the *Matn1-4^−/−^* mice was accompanied by increased collagen fibril diameter (Figure 8A,B), confirming our similar results obtained on the growth plate cartilage, and changes in the nanomechanical properties of the knee joint cartilage. Nano-scale indentation-type AFM recorded the typical bimodal stiffness distribution of the articular cartilage (representing proteoglycans and collagens) [71,72] and showed higher stiffness of both macromolecular assemblies at the superficial zone of the articular cartilage in *Matn1-4^−/−^* mice compared to control already at 4 months of age before any histological sign of cartilage degeneration (Figure 8C). As the stiffness of the middle and deep zones were in the normal range at this age, it seems that matrilin-deficiency primarily affects ECM biomechanics in the outermost zone where the collagen fibrils are oriented parallel to the surface. Of note, a recent study found increased elastic moduli at the articular cartilage surface in *Matn1^−/−^* mice [34]. Furthermore, stiffening of the articular cartilage before the onset of OA has been also recently observed in hypomorphic aggrecan mutant mice [71]. When OA is histologically visible in one-year old *Matn1-4^−/−^* mice, the superficial and middle zones exhibited a reduced Young’s modulus for both proteoglycans and collagens (Figure 8D), probably reflecting the advanced deterioration of the ECM [73]. The increased stiffness in the deep zone may be the consequence of a mechanical adaptation mechanism to the weakened upper zones. Importantly, the lack of matrilins did not influence GAG release in IL-1α stimulated hip explant culture (Figure 9), therefore reasonable to speculate that matrilins protect against OA through biomechanical stabilization of the articular cartilage ECM.

Based on mouse models, the participation of the individual matrilins in OA is controversial. Spontaneous articular cartilage degradation was observed in 1 year old *Matn3^−/−^* mice generated by Chen and his colleagues [35], while *Matn3^−/−^* and *Matn1^−/−^*/*Matn3^−/−^* mice established in our laboratory showed no obvious signs of accelerated articular cartilage degradation in aged animals [32,36]. Similarly, no OA-related phenotype was reported in mouse strains carrying a human *MATN3* MED mutation in wild-type [74] or in *Matn1* null background [75]. Interestingly, a recent study demonstrated that the microRNA miR-483-5p targeted *Matn3* and *Timp2* (tissue inhibitor of metalloproteinase 2), which in turn accelerated articular cartilage degradation in mice with experimental OA [76]. Age-related, spontaneous OA has not been reported in mice lacking MATN1 [31,33] or MATN2 [12]. However, more severe articular cartilage degeneration was observed after surgical destabilization of the medial meniscus in *Matn1^−/−^* mice compared to wild-type control [34]. Since the role of MATN4 in OA has not been investigated, we have also evaluated spontaneous articular cartilage degradation in aged *Matn4^−/−^* mice. MATN4 deficiency had a dramatic effect on the joint by exacerbating OA-like erosion of the articular cartilage at a similar level as we observed in the *Matn1-4^−/−^* mice (Figure 10). Consistent with the observation in the growth plate, we have also found a prominent upregulation of MATN2 expression in the MATN4 deficient articular cartilage (Figure 10C) which, however, was unable to compensate for the lack of MATN4. These findings indicate for the first time that MATN4 is essential to maintain the integrity of the articular cartilage and protect the joints against age-associated osteoarthritis.

The most surprising phenotype what we have observed in the *Matn1-4^−/−^* mice was the patterning defect of the axial skeleton manifested by the highly penetrant (nearly 90%) transition of the L6 vertebra into a sacral identity (Figure 2B–D and Table 1). Disturbance of the vertebral column specification at the lumbosacral junction was also present in *Matn1-4^+/−^* mice demonstrated by partial (asymmetric) or complete sacralization of L6 in two-thirds of the investigated animals (*n* = 6). Congenital skeletal anomalies including sacralization of 6th lumbar vertebra (or 26th pre-caudal vertebra) are common in some inbred strains including the strain 129 [77,78]. Depending on environmental factors, the three vertebral types (L6, L6 to S1, asymmetric L6 to S1) occur about the same frequency in this strain [40,77], and the proportion of the normal L6 identity is significantly increased when 129 animals were raised in 129 x C57BL hybrid females upon ovary transplantation [78]. As we have not observed patterning defects of the vertebral columns in previous single or double knockout matrilin mutant mice [12,31,32,36] maintained on the mixed C57BL/6 × 129/Sv genetic background, and the *Matn1-4^−/−^* mice do not display other skeletal abnormalities of the 129 strain (e.g., accessory sternebrae), we are convinced that the phenotype is the consequence of the lack of all matrilins in the axial skeleton. The molecular basis of how matrilins regulate vertebral specification is not clear and warrant further studies. Interestingly, it has been recently shown that mice lacking the nucleus accumbens-associated protein 1 (NAC1) also exhibit the L6 sacralization phenotype accompanied by the reduced mRNA expression of matrilins in *Nacc1^−/−^* chondrocytes, which is especially significant in case of *Matn3* and *Matn4* [79]. Furthermore, all matrilin family members are expressed in the developing vertebral bodies [12,13], and MATN1 and MATN3 have been implicated in chondrogenic differentiation in vitro [80].

## 4. Materials and Methods

### 4.1. Knockout Mice

Outbred mice (C57BL/6 × 129/Sv) knockout for matrilin-1 (*Matn1^−/−^*), matrilin-2 (*Matn2^−/−^*), matrilin-3 (*Matn3^−/−^*), and matrilin-4 (*Matn 4^−/−^*) were previously generated in our laboratory [12,31,32,37]. Double knockout mice for matrilin-1 and -3 (*Matn1^−/−^/Matn3^−/−^*) [36], matrilin-2 and -3 (*Matn2^−/−^/Matn3^−/−^*), triple knockout mice lacking matrilin-1, -2, and -3 (*Matn1-3^−/−^*), and quadruple knockout mice lacking all matrilins (*Matn1-4^−/−^*) [37] were generated by intercrossing single and multiple matrilin deficient mice. Wild-type littermates were used as control for single and double knockout mice. Triple and quadruple knockout mice were maintained in homozygous mutant breeding. Age-matched wild-type non-littermates on the same C57BL/6 x 129/Sv background were used as control for the *Matn1-4^−/−^* and *Matn1-3^−/−^* mice. Mice were kept under 12 h light/dark cycle, constant temperature, in individually ventilated cages in the Central Animal Facility at the Medical Faculty of the Ludwig-Maximilians-University. Mice were housed in groups of 2–5 per cage and received food and water *ad libitium*. The handling and breeding of all mouse strains have been approved by the government of Upper Bavaria (Application number: 55.2-1-54-2532-15-2016).

### 4.2. Human Samples

Human tibial plateau were obtained from patients undergoing total knee arthroplasty after written consent according to the ethical approval no. 238-15. The whole tissue explant was collected in phosphate buffered saline (PBS) pH 7.4 in the operation room of the Schön Klinik (Munich, Germany) and immediately delivered to our laboratory. Afterwards, cylindrical osteochondral plugs from differently degenerated or non-degenerated areas of the plateau were harvested with the aid of a 7 mm diameter trephine drill, washed once in PBS and fixed in 4% paraformaldehyde (PFA)/PBS overnight at 4 °C. Osteochondral plugs were decalcified in 10% formic acid/dH_2_O for 3 days at RT. After that, plugs were thoroughly washed in PBS, immersed in 20% sucrose/PBS for 24 h at 4 °C and embedded in Tissue-Tek cryomedia (Sakura Finetek, Alphen aan den Rijn, The Netherlands) and gradually frozen on a chilled copper plate placed on dry ice. Sagittal sections of 10 µm were cut using a cryotome (HM500 cryostat, Thermo Fischer Scientific, Waltham, MA, USA) and collected on Superfrost Plus glass slides (Thermo Fischer Scientific, Waltham, MA, USA).

### 4.3. Antibodies

For immunohistochemistry and Western blots, the following primary antibodies were used: Rabbit polyclonal antibodies against matrilin-1, matrilin-2, matrilin-3, matrilin-4, cartilage oligomeric protein (COMP) and collagen VI were described previously [36]. Antibodies against collagen IX were gifts of Susanne Grässel (University of Regensburg, Germany) and Frank Zaucke (Orthopaedic University Hospital Friedrichsheim, Germany). Rabbit polyclonal anti-aggrecan antibody (ab#1031) was obtained from Merck Millipore (Billerica, MA, USA); mouse monoclonal anti-collagen II antibody (CIIC1) was purchased from the Developmental Studies Hybridoma Bank (Iowa, IL, USA); and the rabbit monoclonal antibodies specific for SMAD-1 (D59D7, #6944) and for phospho-SMAD-1/5/8 (D5B10, #13820) were obtained from Cell Signaling Technology (Beverly, MA, USA). Primary antibodies were diluted 1:400 for immunohistochemistry and 1:1000 for immunoblotting.

### 4.4. Whole-Mount Skeletal Staining and X-ray Analysis

For skeletal staining, 2-day and 4-week old mice were euthanized with carbon dioxide. De-skinned and eviscerated specimens were fixed in 95% ethanol for 3 days and transferred into acetone for additional 2 days. The skeleton was stained with 0.6% Alcian Blue (for cartilage) and 0.02% Alizarin Red (for bone) (both Sigma-Aldrich, St. Louis, MO, USA) in 90% ethanol and 5% acetic acid for 3 days in a 37 °C incubator with continuous shaking. Samples were cleared by incubation in descending potassium hydroxide and ascending glycerol solutions, and were finally preserved in 100% glycerol. To determine the length of the long bones, the humerus, the femur, and the tibia were dissected from five control and five *Matn1-4^−/−^* mice, photographed with a Stemi 1000 stereo microscope (Carl Zeiss, Jena, Germany) and measured with the ZEN software (Carl Zeiss, Jena, Germany).

For X-ray analysis, 4-week, 4-month, and 1-year old mice were euthanized, and radiographs were taken using a sealed X-ray cabinet (FAXITRON 43855 A) at 35 kV, 2 mA, and 2 s exposure time. Total body length (from the snout to the end of the tail) and the length of the skeletal elements (tibia, femur, and humerus) were analyzed with the distance measurement plug-in of the syngo Imaging XS-VA60B software (Siemens, Erlangen, Germany).

### 4.5. Histology, Immunohistochemistry and In Situ Hybridization

Mouse limbs were dissected from 15.5 and 18 day-old embryos (E15.5 and E18), and from newborn, 2-day (P2), 2-week, 4-week, 2-month, 4-month, 8-month, and 15–24 month old animals. The specimens were routinely fixed in 4% PFA/PBS at 4 °C for 12–24 h. Additionally, forelimbs were fixed in 95% ethanol and 1% acetic acid for immunohistochemical analysis. Samples from 2 weeks of age were decalcified in 15% ethylenediaminetetraacetic acid (EDTA) (Sigma-Aldrich, St. Louis, MO, USA) dissolved in PBS (pH 8.0) for 1–3 weeks. Specimens were processed either for standard paraffin or for cryo embedding. All the embedded tissues were cut into 8-µm thick sections using a microtome or cryotome. For routine histology, the sections were stained with hematoxylin and eosin (HE), Safranin orange and fast green (SO/FG), and Safranin orange-von Kossa (SO/vK) according to the standard protocols. Morphometric analyses of the growth plate zones were performed as described previously [81]. TRAP (tartrate resistant acid phosphatase) staining to visualize chondroclasts/osteoclasts at the chondro-osseous junction was performed with the leukocyte acid phosphatase kit (Sigma-Aldrich, St. Louis, MO, USA) according to manufacturer instructions. To assess articular cartilage degeneration on HE stained sections, a histological grading score for structural alteration was applied as follow: 0-normal articular cartilage; 1-surface irregularities; 2-cleft to transition zone; 3-cleft to radial zone; 4-cleft extending to calcified zone; 5-exposure of subchondral bone [36].

For immunohistochemistry, paraffin sections were rehydrated in descending ethanol series, rinsed in PBS and treated with bovine testicular hyaluronidase (Sigma-Aldrich, St. Louis, MO, USA) (2 mg/mL in PBS, pH 5.0) for 30 min at 37 °C to facilitate antibody penetration. Primary antibodies were incubated overnight at 4 °C and the subsequent immunohistochemical detection was performed using the corresponding Vectastain ABC Elite kit (Vector Laboratories, Burlingame, CA, USA) and 3,3-diaminobenzidine (DAB, Sigma-Aldrich, St. Louis, MO, USA) as chromogenic substrate.

For non-radioactive in situ hybridization, deparaffinized sections were rinsed in Tris-buffered saline (TBS) pH 7.5, acetylated with 0.25% acetic anhydride (pH 8.0) for 10 min, rinsed in TBS and dehydrated in ethanol. Sections were hybridized at 70 °C overnight with digoxigenin (DIG)-UTP-labelled antisense riboprobes specific for mouse collagen II (*Col2a1*), collagen X (*Col10a1*), indian hedgehog (*Ihh*), parathyroid hormone/parathyroid hormone-related peptide receptor (*Ppr*) and matrix metalloproteinase-13 (*Mmp-13*) as previously described [32]. After hybridization and subsequent washing in 2 X sodium citrate-chloride buffer (SSC) at 70 °C for 30 min, the sections were blocked with 2% goat serum for 1 h and incubated with a 1:500 diluted alkaline phosphatase-coupled anti-DIG antibody (Roche, Penzberg, Germany) for 2 h. Hybridization signals were detected using the p-nitroblue tetrazolium chloride/5-bromo-4-chloro-3-indolyl phosphate (NBT/BCIP) solution (Roche, Penzberg, Germany) according to the recommendation of the manufacturer. Brightfield microscopy images were acquired with an AxioObserver microscope (Carl Zeiss, Jena, Germany).

### 4.6. Cell Proliferation and Cell Death Assays

In vivo chondrocyte proliferation was analysed using the 5-bromo-2´-deoxyuridine (BrdU) incorporation assay as described previously [32]. Briefly, mice were injected intraperitoneally (50 μg/g of body weight) with BrdU solution (10 mg/mL in PBS, pH 7.4). After 2 h, the animals were sacrificed, knee samples were dissected and embedded into paraffin. Deparaffinized sections were treated with 2 M HCl for 30 min, washed in PBS, and incubated with a peroxidase-conjugated antibody against BrdU (Roche, Penzberg, Germany). Detection of proliferative cells in the S phase of the cell cycle was performed with DAB as chromogen substrate. Analysis of apoptotic chondrocytes was carried out using the TUNEL assay according to the manufacturer’s instructions (In Situ Cell Death Detection Kit, Roche, Penzberg, Germany).

### 4.7. Protein Extraction and Western Blotting

Knee joint cartilage was dissected from newborn and 4-week old *Matn1-4^−/−^* and control mice (*n* = 10). Specimens were weighed, cut into small pieces, and incubated in ten volumes of chilled extraction buffer I (0.15 M NaCl, 50 mM Tris, pH 7.4) overnight at 4 °C with continuous stirring. Next day, the mixture was centrifuged at 14,000 rpm for 1 h and the supernatant was stored at −20 °C, and the obtained pellet was incubated as above in buffer II (1 M NaCl, 10 mM EDTA, 50 mM Tris, pH 7.4) and subsequently in buffer III (4 M guanidine hydrochloride, 10 mM EDTA, 50 mM Tris, pH 7.4). All extraction buffers contained EDTA-free proteinase inhibitor cocktail (Roche, Penzberg, Germany). Aliquots (100 µL) of all extracts were precipitated with 96% ethanol and the pellets were processed and re-suspended in non-reducing SDS-PAGE (sodium dodecyl sulfate–polyacrylamide gel electrophoresis) sample buffer as described previously [32]. Samples were applied to 4–15% pre-casted SDS-PAGE gels (Bio-Rad, Berkeley, CA, USA) and electrophoresis was performed using a Bio-Rad apparatus. For Western blotting, proteins were transferred to a PVDF membrane (GE Healthcare, Chicago, USA) and incubated with primary antibodies diluted either in 5% skim milk (Sigma-Aldrich, St. Louis, MO, USA) or 5% bovine serum albumin (BSA) (Sigma-Aldrich, St. Louis, MO, USA) in 1X TBST (0.5% Tween 20 in TBS). Bound antibodies were detected by HRP-conjugated secondary antibodies, and the signal was developed using Luminata ECL Forte (Merck Millipore) and acquired on the GE Healthcare imaging system ImageQuant LAS 4000.

Primary chondrocytes were isolated from newborn ribcage as described [71]. Single cell suspensions were lysed in RIPA buffer with phosphatase (PhosSTOP, Roche, Penzberg, Germany) and protease inhibitors. Total protein was normalized using the BCA assay (Themo Scientific, Waltham, MA, USA) and equal amounts of protein were subjected to 10% SDS-PAGE, transferred to PVDF membrane and imaged as above.

### 4.8. Explant Culture

Femoral heads were dissected from 4-week-old control and *Matn1-4^−/−^* mice and cultured in serum-free Dulbecco’s modified Eagle’s medium (DMEM) supplemented with streptomycin-penicillin for 4 days at 37 °C and 5% CO_2_ in the absence or presence of the catabolic cytokine IL-1α (10 ng/mL) (R&D Systems, Minneapolis, MN, USA). After the culture period, femoral heads were fixed in 4% PFA, cryo-embedded, cut, and stained with SO to examine the loss of proteoglycans. Conditioned culture medium was also collected and analyzed for released sulfated glycosaminoglycan (sGAG) using the Blyscan B1000 GAG assay (Biocolor Ltd., Carrickfergus, UK) according to the instructions of the manufacturers.

### 4.9. Atomic Force Microscopy (AFM) 

Knee joints from 2-week, 4-month, and 1-year-old control and *Matn1-4^−/−^* animals were dissected and immediately immersed into Tissue-Tek and snap frozen in a liquid nitrogen-cooled bath of isopentane. Sagittal sections (30 μm) of three animals per genotype were cut using two supportive tapes (one double adhesive and an adhesive) and a cryostat, and placed on Super Frost Plus glass slides as previously described [82]. Sections were stored at −20 °C until the analysis. AFM imaging and indentation measurements were carried out on sections equilibrated at room temperature using the NanoWizard 1 AFM (JPK Instruments, Berlin, Germany) in combination with an inverse optical microscope (Axiovert 200, Carl Zeiss, Göttingen, Germany) as previously described [82]. Briefly, for the growth plate measurements the Young’s modulus was determined at each indentation position by fitting a modified Hertz model (pyramidal indenter) to the respective approach curve, using the JPK data processing software (versions 4.2.20 and 5.0.96; JPK Instruments). The contact point was determined manually for each force curve, and the fit range was limited to a maximum indentation depth of 500 nm. For the articular cartilage measurements, the contact point was determined by fitting the modified Hertz model to the entire force range first and then using this fix contact point to fit only the first 500 nm indentation depth. To generate histograms and fitting Gaussian distributions, the data analysis software Origin 8.0 (OriginLab Corporation, Northampton, MA, USA) and Igor Pro 6.37 (Wavemetrix, London, UK) were used for growth plate and articular cartilage data, respectively. AFM imaging of the middle and deep zone of the articular cartilage was performed on 20 μm thick, PFA-fixed sections.

### 4.10. Statistical Analysis

Data are presented as mean ± SD (standard deviation). Statistical significance was assessed using the Mann-Whitney *U* test, *p* values less than 0.05 were considered significant. Histology data are representative of a minimum of six animals for each group.

## 5. Conclusions

In summary, our data imply that the matrilin family of adaptor proteins is dispensable for the growth of the cartilaginous skeleton, but required for vertebral column specification and articular cartilage stability. Biochemical analyses of cartilages from various matrilin-deficient mouse lines showed compensation among the family members, and indicated that the lack of all four matrilins moderately changes the extractability of binding partners. Atomic force microscopy revealed that matrilins control collagen fibril diameters in the cartilage ECM and modulate the stiffness of the articular cartilage. *Matn1-4^−/−^* and *Matn4^−/−^* mice develop similar age-associated osteoarthritis suggesting a novel function of matrilin-4 for preventing articular cartilage degradation in the murine knee joint.

## Figures and Tables

**Table 1:** Axial skeletal phenotypes in the respective genotypes.

Lumbosacral Pattern	Control	*Matn1-4^+/−^*	*Matn1-4^−/−^*
	(*n* = 45)	(*n* = 7)	(*n* = 40)
L6/S4	42 (93.3%)	3 (42.8%)	6 (12.5%)
L6*/S4	0	2 (28.6%)	0
L5/S5	3 (6.7%)	2 (28.6%)	34 (87.5%)

## Abbreviations

ACAN	Aggrecan
AFM	Atomic force microscopy
BMP-2	Bone morphogenetic protein-2
BrdU	Bromodeoxyuridine
COL	Collagen
COMP	Cartilage oligomeric matrix protein
ECM	Extracellular matrix
EDTA	Ethylenediaminetetracetic acid
GAG	Glycosaminoglycan
HE	Hematoxylin and eosin
IL-1	Interleukin-1
ITM	Interterritorial matrix
MATN	Matrilin
MED	Multiple epiphyseal dysplasia
OA	Osteoarthritis
PBS	Phosphate buffered saline
PFA	Paraformaldehyde
P2	Postnatal day 2
SDS-PAGE	Sodium dodecyl sulfate-polyacrylamide gel electrophoresis
SEMD	Spondylo-meta-epiphyseal dysplasia
TRAP	Tartrate-resistant acid phosphatase
TUNEL	Terminal deoxynucleotidyl transferase dUTP nick end labeling
